# Supplementary material for: Are carbon emissions trading and green financial instruments synergistic? -Comprehensive quantitative research based on content analysis
Source: PLoS One. 2024 Mar 7;19(3):e0298601. doi: 10.1371/journal.pone.0298601 (PMC10919601; doi:10.1371/journal.pone.0298601)
Supplement: S1 Data — (ZIP) [file pone.0298601.s001.zip › Data/地级市Z_F工作报告.zip!/┴╔─■/░░╔╜╩╨/└┤╫╘╣1⁄2╓┌║┼í╛┬φ┐╦╩2╛▌═°í┐_2009.docx]

各位代表：

现在，我代表市人民政府向大会报告工作，请予审议，并请市政协各位委员提出意见。

一、2008年政府工作回顾

2008年是极不寻常的一年，是鞍山战胜各种困难，实现大发展、快发展的一年。在市委的领导下，在市人大、市政协大力支持下，市政府认真贯彻落实科学发展观，按照市委十届四次全会提出的“围绕一个核心，实现两个融入，推进三大任务”总体要求，一手抓发展、一手抓民生，一手抓城市、一手抓农村，一手抓空间、一手抓项目，全面超额完成市十四届人大一次会议确定的工作目标，全市经济社会发展呈现强劲态势，取得历史性跨越。预计，全年实现地区生产总值1608亿元，增长18.3%；实现规模以上工业增加值670亿元，增长17.5%；实现第三产业增加值628亿元，增长19.2%；完成地方财政收入160亿元，增长55%，其中一般预算收入首次超过百亿元，完成100.01亿元，增长34.2%；全市银行业金融机构存款余额达到1350亿元，增长17.4%，贷款余额首次突破800亿元，增长26%，实现利润16亿元，增长15.5%；完成全社会固定资产投资700亿元，增长46.8%；实际利用外商直接投资5.8亿美元，增长1.1倍；引进内资455亿元，增长30%；外贸出口31.5亿美元，增长31%；实现社会消费品零售总额367亿元，增长23%；城市人均可支配收入达到15450元，农民人均纯收入达到7255元，分别增长20.2%和18.5%。

过去一年，我们主要做了八个方面工作：

（一）积极推进“两个融入”，鞍山发展新格局加速形成。编制完成鞍海经济带发展规划，全面推进鞍海经济带建设，鞍山融入辽宁沿海经济带和沈阳经济区步伐明显加快。

城市发展格局正在形成。按照西部产业带、中部生活带、东部生态带发展方向，完成土地利用规划调整，城市总体规划修编取得阶段性成果。城市西部及北部集中发展工业，新上工业项目进入园区，城区企业加快向园区搬迁，实现了工业进园区发展。东部建设生态功能区，把千山作为城市资源，遵循“大规模保护、小规模开发”原则，启动了企业搬迁和居民动迁工作，重点发展文化旅游产业项目。南部规划建设新城区，坚持规划先行，完善了新城区控制性详细规划。中心城区大力发展现代服务业和房地产业，加快推进主街主路两侧及危旧房改造，实现了商业进城区和民居进小区发展。全年拓展发展空间25平方公里，搬迁企业240家，拆动迁居民1.6万户。

产业发展格局正在形成。工业区建设加速推进。腾达通海产业大道建至旧腾路，形成了包括达道湾、灵山、高新区（西区）、宁远、汤岗子农高区在内的鞍山工业主体功能区。达道湾工业区成为沈阳经济区重点起步区，基础设施更加完善，开工建设项目152个，建成投产企业72家。服务业布局全面推进。按照块状化、带状化、网格化、点状化商业布局要求，重点打造八条精品商业带，建设两大物流园区，形成九大商贸集聚区。设施农业加速发展。鞍羊线、鞍营线、张庄线三条设施农业示范带全面建设，鞍羊线成为现代农业的集中带、农民致富的黄金带、新农村建设的示范带。

县域发展格局正在形成。海城打破区划界限，规划建设五大经济区，形成“一带三区八园”工业发展新格局，海城经济开发区和腾鳌经济开发区建设取得重大突破。台安形成“一带两区”工业发展新格局，台安工业区提档升位，成为县域经济先导区。岫岩形成“一带三区”工业发展新格局，偏岭工业区和兴隆工业区项目承载能力全面提升。全市16个县域工业区规划开发面积由39平方公里增加到59平方公里。海西新区、腾鳌镇等县城和中心镇改造全面推进，县域服务业和房地产业加速发展。

（二）深入开展项目升级年活动，产业结构调整迈出新步伐。坚持以项目为核心，全力推进项目引进和开工建设，项目规模、质量、效益、结构全面升级。

全方位开展招商引资工作，项目数量取得较大突破。全面引进中省直、本地民营、外埠民营和境外资本投资鞍山。全市“四个一批”项目总数达到2016个，总投资3085亿元。新开工项目1060个，竣工投产项目405个。新增规模以上工业企业400家，总数突破1700家；新增规模以上商业企业105家，总数超过800家。

鞍钢等中省直及民营企业加大投资，百亿元项目取得较大突破。鞍钢克服金融危机影响，加大投资力度，实现平稳较快增长。30万吨有取向硅钢、60万吨煤焦油深加工、70万吨合金线材、15万吨苯加氢等一批重大项目开工建设。全年完成固定资产投资200亿元，增长42.9%；实现工业增加值380亿元，增长8%；实现税收114.3亿元，增长18%；形成地方财政一般预算收入33.1亿元，增长15.2%。鞍钢等中省直及民营企业为全市经济社会发展做出重大贡献。全市销售收入超百亿元项目达到26个，比上年增加15个。中船重工200万吨船板加工、中航科技产业基地、中冶东建管材及风电塔基、宝得150万吨H型钢、后英菱镁高科技产业园等18个项目开工建设，部分项目形成生产能力。浙江普及300万吨重油制烯烃、国电集团8×300MW热电机组等8个项目完成规划和选址。

大力发展产业集群，“一个基地、四大产业”项目取得较大突破。全年引进和实施集群项目568个，总投资1555亿元。精特钢和钢材深加工产业集群做大做强。新上项目146个，总投资535亿元，钢材本地加工率由上年12%提高到20%。冶金装备制造及自动化产业集群不断壮大。新上项目181个，总投资230亿元，装备制造业占全市工业比重由上年9%提高到12%。煤焦油、石油化工新材料及深加工产业集群异军突起。新上项目109个，总投资405亿元，项目建成后年销售收入将超千亿元，成为鞍山未来重要的支柱产业。菱镁、滑石等矿产品深加工产业集群加速形成。新上项目132个，总投资385亿元，全市矿产品深加工率由上年40%提高到47%。

加速发展服务业和房地产业，优化项目结构取得较大突破。服务业实现大发展、快发展。东北商贸城和北方钢铁大市场建设取得重大进展，中豪威尔东北化工建材城、凯兴物流冷冻大市场等5个大型专业批发市场和北方金属交易中心、瑞盛汽配城等22个项目开工建设。解放西路汽车贸易产业带形成规模，新发展汽车专营店33家。胜利路、解放路和南建国路精品商业带迅猛发展，引进了法国宜必思和皇冠假日等大型酒店、家乐福和华润万家等大型超市、大商新玛特购物广场和兴隆大家庭、时代商厦等大型精品百货。千山东路文化旅游产业带启动建设，洽谈推进了千山海洋大世界、热岛玉温泉、穹幕影城等文化旅游产业项目。全年开工建设服务业项目177个，总投资188亿元；服务业实现税收70亿元，增长40%。全年接待境内外旅游者1545万人次，增长25.4%；实现旅游综合收入112亿元，增长30.2%。房地产业高歌猛进。全年开发万科城、大商尚城、新景世纪城、香港新世界等房地产项目148个，开工建设面积达到1290万平方米，增长1倍；完成投资130亿元，增长1.2倍；销售房屋247万平方米，实现销售额72亿元，均增长1倍以上。高新技术产业加快发展。以高新区为载体，省级高新技术企业发展到204家，实现高新技术产品产值450亿元，增长27.2%。

（三）大力发展县域经济，新农村建设登上新台阶。全年市本级投入“三农”资金9亿元，比上年增长50%。

县域经济实现大发展。县域地区生产总值完成737.5亿元，增长28.5%；县域财政一般预算收入完成25.8亿元，增长38.8%；县域规模以上工业增加值完成224.5亿元，增长30.9%。全年粮食总产量达到143万吨，总产和单产均创历史最高水平。

县域主导产业加速发展。海城重点打造菱镁深加工、滑石方解石添加剂和钢铁深加工产业集群；台安重点打造木制品深加工、建筑起重机械产业集群；岫岩重点打造矿产品深加工、中药材深加工产业集群。全年县域工业新上项目444个，比上年增加148个，总投资204亿元；县域规模以上工业企业总数达到1307家。

设施农业实现迅猛发展。继续加大对设施农业支持力度，全市新发展温室大棚5.9万栋、标准化养殖小区221个，新增设施农业23万亩，总面积达到73万亩，占农业比重由上年15%提高到21%。通过发展设施农业，农民人均增收500元以上。

（四）高度重视节能减排，转变发展方式取得新成效。积极推进节能减排、环境整治和安全生产，经济发展体现好字优先。

节能减排取得成效。全年推进重点节能降耗技术改造项目36个，对193家高能耗企业实行差别电价，累计关停高能耗高污染企业310家。全市万元GDP综合能耗下降5.3%。完成污染减排项目157项，削减了二氧化硫排放总量。

生态环境加速治理。开展辽河流域水污染防治，实施水环境治理工程206项。规划建设10个污水处理厂，已有5个开工建设，第二污水处理厂二期建成使用，国控断面化学需氧量年均浓度下降17.7%，达到地表水四类标准。对10平方公里排岩场及尾矿库实施了复垦和抑尘工程，全年城区空气环境质量达到国家二级标准天数首次超过300天。全年植树1809万株，造林11.4万亩。启动台安西北部沙化治理工程，造林2.61万亩。完成集体林产权制度主体改革。

安全生产工作全面加强。组织实施安全生产和消防安全“五大战役”，不间断开展隐患大排查大整改行动，全年排查和消除各类安全隐患8490个。6家烟花爆竹生产企业全部退出市场，不合格危险化学品生产企业关停整改，安全不达标非煤矿山和尾矿库全部关闭。

（五）深入开展文明城市创建活动，城乡建设和管理水平全面提升。继续加大对城乡建设和改造的投资力度，实施重点项目190个。

基础设施不断完善。城市一环线全面开通，二环线开工建设；改造胜利路、西解放路、绿化街、湖南街等17条主次干道，同步改造和铺设地下管线70.8公里；深营路东线隧道、站前过街通道及虹桥改造工程顺利实施；改造县乡村三级公路370公里，实现村通油路78公里；哈大快客铁路和丹海高速公路加快建设。达道湾500千伏输变电工程投入运行，新建和续建220千伏变电所9座、66千伏变电所10座。引汤入鞍二期供水工程、大伙房水库引水工程、地方与鞍钢合作煤制气项目加速推进。沈阳桃仙机场鞍山候机楼投入使用，启动了腾鳌机场和航站楼民用改造工程。新增城区停车场50处、社区“两站四室”40个、公厕70个。

城乡环境全面改善。南沙河、杨柳河、运粮河及海城河综合整治工程全面实施，正在建设胜利湖、杨柳湖等城市水系节点。完成二一九、孟泰、永乐公园二期改造，新建湖南公园、劳动公园和东解放路绿地公园，新增城市绿地100万平方米。改造居民小区30个、街巷路15条，安装和更新路灯1073基。绿化村屯800个。

城乡管理不断加强。全面整顿马路市场，新建农贸市场25个，取缔占道市场23处。拆除违章建筑767处，拆除违章及破损的广告牌匾和灯箱8000多个。建成辽宁科技大学过街天桥。取消了市区出租车、运输车等车辆过桥费项目，撤销了鞍山城和宁远收费站。实施道路交通整治百日行动，城市交通状况有所改善。以鞍羊线、鞍海线、大盘线沿线村屯为重点，全面加强了村容村貌环境整治。

（六）切实加强社会建设，各项社会事业全面进步。全年投入社会建设资金超过7亿元，是历史上最多的一年。

教育事业均衡发展。新建10所农村九年一贯制学校，改造城区20所薄弱中小学校，免除城区8.5万名学生义务教育阶段学杂费，双高普九工作走在全省前列。在全国率先探索实行高中免费教育，7个县（市）区各设1所免费高中。启动了鞍山职教城建设。

卫生事业加快发展。进一步完善公共卫生服务体系、社区卫生服务体系和农村卫生服务体系。建成中心医院住院大楼和汤岗子医院新治疗中心。建立社区卫生服务中心26个、社区卫生服务站57个。改造22所乡镇卫生院，为973个村卫生所配备基本医疗设备，农村卫生院和村卫生所装备全部达到国家规定标准。

文体事业全面发展。改造建设了市博物馆、群众艺术馆、档案馆、图书馆和青少年活动中心，建设200个农村图书室，现代农民远程科技培训视频网络覆盖所有行政村。全年组织大型文化广场演出100余场，免费为农民及农民工演出158场，丰富了城乡群众业余文化生活。为每个行政村配备了健身器材，为每个社区配备了乒乓球台。31所市区中小学操场清晨向市民开放。圆满完成奥运火炬传递和安保任务。鞍山籍体育健儿在北京奥运会上获得3枚金牌、2枚铜牌，金牌数和奖牌数位列全省第一。我市残疾人运动员在北京残奥会上，获得1枚金牌、2枚铜牌。

社会管理全面加强。全市应急联动体系不断完善。强化了对食品药品的市场监管。居民消费价格指数低于全省、全国平均水平。全面规范了住宅小区物业管理。深入开展“五五普法”工作。积极推进平安钢都建设，严厉打击各类刑事犯罪，加强社会治安综合治理，努力解决群众信访问题，和谐鞍山建设迈出新步伐。积极支援四川抗震救灾，捐助款物1.3亿元，派出救援队和医疗队200人次。

宗教、侨务政策全面落实，全市各民族团结和谐，老龄、妇女儿童、人口与计划生育、残疾人工作不断加强，人防、国防后备力量建设工作继续强化，新闻出版、广播电视、外事、对台、统计、史志、档案、气象、地震、接待等工作都有新成绩。

（七）努力解决民生问题，城乡居民生活水平有新提高。全年为城乡群众办的20件实事和重点民生工程较好完成。

解决就业有新成绩。全年实现实名制就业13万人。通过扩大就业，城镇登记失业率由4.3%下降到3.8%，城市低保人员由13.4万人降至10.5万人，减少2.9万人。建成大学生就业中心和创业基地，大中专毕业生就业率超过90%。部队转业干部全部安置，为退役士兵提供免费就业培训。建成市劳动力大厦。

社会保障有新进展。推进社会保险应保尽保。全年征收保险金40亿元，增长19.5%。其中，城镇企业职工养老参保55.45万人，新增8.6万人，征收保险金12.7亿元，增长27.6%；职工基本医疗参保97.3万人，新增6.03万人，征收保险金9.2亿元，增长9%；失业、工伤、生育保险新增参保人数和基金征缴均增长30%以上。推进医疗保险全覆盖。城镇居民基本医疗参保21万人；农村新型合作医疗参合率达到96.8%，筹资标准由50元提高到100元，参合农民报销额提高1倍；为城乡困难群众免费体检4.7万人次。完善社会救助制度。建立了农村困难居民医疗救助制度和城乡低保边缘户救助制度，开展扶贫帮困活动，保障了困难群众的基本生活。

解决安居有新突破。建立起多层次住房保障体系，解决不同收入家庭住房问题。开工建设回迁房295万平方米、低价房1000套、廉租房500套，回迁居民1.26万户。帮助3000户农村困难家庭解决住房问题。开工建设了军人之家和劳模大厦。

生活条件有新改善。供暖设施改造年活动效果良好。政府和企业共投资4.4亿元，维修改造锅炉58台，更新改造管网50公里，新发展供暖面积150万平方米，实施分户改造105万平方米，供暖质量得到改善。新购城市公交车110辆。新建农村乡镇客运站4个。解决了48万农民饮水安全问题。新建50个农村生物质燃气站。改造农村敬老院22所，全市农村敬老院达到省级标准。

（八）全面加强政府自身建设，依法行政水平不断提高。

民主法制建设全面加强。积极向人大及其常委会报告工作，与人民政协民主协商。自觉接受人大及其常委会的依法监督、人民政协的民主监督、人民群众的社会监督。认真执行人大及其常委会的决议，积极采纳政协意见，人大代表建议和政协委员提案办结率、见面率和满意率达到100%。

政府制度建设不断完善。全面规范政府经济活动。国有土地出让全部实行公开挂牌交易，政府采购和投资建设项目全部公开招标，引入被监督机制，创新实行了代购代建制，全年市本级政府采购项目节余资金10%，投资建设项目招标节余资金13%。全面优化投资发展环境。大力削减行政审批事项和行政收费，行政审批事项由449项减少到104项，行政收费项目减少457项，年减少收费额1.7亿元；对现有行政收费全部实行定值管理，投资建设项目取消行政罚款。全面推行政府信息公开，保证了广大群众知情权、参与权和监督权。

工作作风进一步改进。进一步加强勤政廉政建设，强化了行政效能监察。通过市区人民来访接待中心、市民投诉电话、市长信箱进社区、市长电子信箱等多渠道听取群众意见、建议和诉求，解决群众反映的实际问题。深入调查研究，坚持现场办公，提高办事效率，有力推进了招商引资、项目服务、园区建设、城市改造、安全生产、为群众办实事等各项工作的落实。

各位代表，鞍山的变化令人振奋，鞍山的发展充满希望。过去一年取得的成绩，得益于市委的正确领导和市人大、市政协的大力支持，得益于鞍钢等所有企业做出的巨大贡献，得益于全市人民的共同努力。在此，我代表市政府向心系鞍山发展的各位代表和委员，向奋战在全市各条战线的广大干部群众，向为鞍山发展做出巨大贡献的鞍钢等中省直单位和各企业，向关心和支持鞍山发展的民主党派、工商联、人民团体、驻鞍部队、武警官兵和老领导、老同志，向所有关心和参与鞍山发展的海内外朋友，表示衷心的感谢！

在肯定取得成绩的同时，我们也清醒地看到鞍山发展中存在的问题和面临的困难。主要是，受世界金融危机影响，钢铁企业生产经营出现较大困难；结构调整需加大推进力度，还缺少在全省乃至全国有影响的标志性、牵动性大项目，现代服务业和县域经济比重仍然偏低；城乡差距仍然较大，城乡一体化建设还需进一步加强；节能减排任务艰巨，能源消耗和主要污染物排放强度仍处于相对较高水平；安全生产、社会治安、信访问题等影响社会稳定因素仍然存在；政府工作需要不断加强和改进。上述问题，我们将在今后工作中认真加以解决。

二、2009年政府工作任务

2009年是世界金融危机持续影响较大的一年，也是鞍山实现大发展、快发展的关键一年。当前，世界金融危机的影响已经由虚拟经济扩散到实体经济、由加工工业扩散到原材料工业、由中小企业扩散到大型企业，发展中的不确定因素增多。国家从扩大内需角度，减免房地产交易税费和取消100多项行政收费，提高所得税起征点，提高养老金和事业单位人员工资标准，这些减税让利政策直接导致财政增支减收。同时，我们也清醒地看到，国家加大基础设施和保障性住房投资力度，支持房地产、汽车、家电等产业发展，将会拉动钢铁产业加速发展。我市钢铁产品成本相对较低，在国际国内市场具有较强竞争力。国家扩大信贷投放，将有效缓解企业资金紧张状况。经过多年持续招商引资上项目，我市形成了一大批新的经济增长点。我们鞍山一定能够克服困难、抓住机遇，在沧海横流中继续更好更快发展，我们350万鞍山人民一定能够创造出更加美好的明天！

新的一年政府工作的总体思路是，全面贯彻党的十七大、十七届三中全会和中央、省、市经济工作会议精神，深入学习实践科学发展观，按照市委确定的“围绕一个核心，实现两个融入，推进三大任务”总体要求，继续坚持一手抓发展、一手抓民生，一手抓城市、一手抓农村，一手抓空间、一手抓项目，促增长、扩内需、调结构、转方式、重民生、保和谐，全面推进经济建设、文化建设、社会建设和城乡一体化建设，努力实现鞍山经济社会大发展、快发展。

综合考虑鞍山发展面临的机遇、现实条件和存在的困难，新一年主要目标是，实现地区生产总值增长15%；地方财政一般预算收入增长15%；实际利用外商直接投资增长20%，引进内资增长20%；全社会固定资产投资增长30%；外贸出口增长10%；社会消费品零售总额增长15%；城市居民人均可支配收入和农民人均纯收入分别增长10%以上；万元GDP综合能耗下降5.3%，主要污染物排放量平均减少5%。

按照上述总体思路和主要目标，重点做好八项工作：

（一）积极抢抓机遇，全力以赴促增长。认真落实国家扩大内需方针，加大投资力度，扩大对外开放，推进鞍山经济社会大发展、快发展。

扩大内需促增长。全年建设项目超过2000个，总投资4000亿元，当年开工建设项目超过1000个，力争实现固定资产投资1000亿元。加大工业投资。重点推进重大项目建设和产业集群发展，占全市投资比重达到53%左右。加大服务业投资。大力发展批发市场、精品商业、文化旅游和房地产业，投资比重提高到30%以上，其中房地产业力争超过20%以上。加大农业投资。完善农业基础设施，大力发展设施农业和农产品深加工产业。加大基础设施投资。重点推进城乡基础设施建设、社会建设、节能减排和环境治理。

全面开放促增长。积极引进境外资本投资鞍山，重点引进世界五百强企业。大力引进南方资本投资鞍山，使鞍山成为南方产业转移的承接地。全力争取更多中省直企业投资鞍山，支持鞍钢等钢铁企业重组壮大，培育新的百亿元项目。鼓励本地企业投资发展。加大对中小企业支持力度，促进企业稳定发展。积极主动争取国债投资项目。

挖掘潜力促增长。抓住国家强化基础设施建设投资的机遇，鼓励企业扩大生产。大力发展重轨、取向硅钢、型材、管材等钢材及深加工产品；发展以工程机械为重点的装备制造产品，抢占国内市场。抓住国家扩大信贷规模的机遇，帮助企业融资。鼓励企业上市，促进银企对接，引导金融机构信贷投向，合理扩大信贷规模，全市银行业金融机构贷款余额争取突破1000亿元。抓住国家扩大民生投资的机遇，积极促进服务业、房地产业和社会事业持续快速健康发展。

（二）继续开展项目升级年活动，大力推进产业升级。以项目升级推进结构调整，全面提升鞍山产业竞争力。

推进项目向园区集中。按照集中式布局、集约化生产、集群化发展要求，加快园区建设。以加速鞍海经济带建设为重点，以腾达通海产业大道为纽带，加快建设达道湾工业区、灵山工业区、高新区、宁远工业区、汤岗子农高区、腾鳌经济开发区、海城经济开发区等十大工业区，全面完善道路、供电、供气、供暖、给排水等基础设施，推进标准化厂房建设，实现项目进园区发展。

推进项目向规模升级。全力推进百亿元项目招商引资、开工建设和竣工投产，全年新增销售收入超百亿元项目10个以上，总数超过36个。开工建设工业项目超过800个，商业项目超过150个，新增规模以上企业500家。

推进项目向主导产业集聚。按照“一个基地、四大产业”结构调整方向，推动产业升级。大力推进钢铁深加工多样化。围绕精特钢，重点发展有取向硅钢、冷热轧不锈钢、轴承用钢。发展硅钢深加工、塔筒、型材及紧固件，为输变电产业配套；发展电镀锌板、不锈钢板、不锈钢与镀锌复合板及深加工，为汽车、家电、食品及环保产业配套；发展船用板材及其他板材深加工，为造船、电气等产业配套；发展重轨、导轨，为铁路、港口、矿山、运输及电梯产业配套，把鞍山建成世界级精品钢生产及钢材深加工配送基地。大力推进冶金装备成套化。依托中冶焦耐、中冶北方、中钢热能和鞍钢设计院的设计研发、总包集成优势，做大做强本地骨干企业，积极引进外埠相关企业，不断完善产业链条，形成矿山设备成套、焦化冶炼设备成套和轧钢设备成套，努力推进由重大装备制造走向设备成套和工程成套，把鞍山打造成冶金装备制造大市。大力推进化工新材料系列化。依托鞍钢60万吨煤焦油，大力发展煤化工。以煤焦油分离出的沥青为原料，依托中钢集团，发展煤系针状焦和核石墨等超高功率电极产品；以煤焦油分离出的蒽油为原料，依托台湾中橡，发展炭黑等产品；以煤焦油分离出的酰油为原料，依托鞍山惠丰化工，发展工业颜料、染料等产品。依托中石油和浙江普及300万吨重油制烯烃等石化资源，大力发展石油化工。以石油分离出的乙烯、丙烯、氯乙酸等为原料，依托尼龙66盐、苯加氢和尼龙6项目，发展塑料原料及制品、化工粘合剂、添加剂、融合剂等产品，将化工新材料及深加工产业打造成鞍山新的支柱产业。大力推进矿产品深加工多元化。利用菱镁资源发展镁耐火材料、镁金属及深加工和镁制建筑材料；利用滑石和方解石资源发展添加剂产业集群，发展涂料、油漆、造纸、化妆品等深加工产品，实现由矿产粗加工制品向精深加工和制成品升级。大力推进光电产业规模化。支持现有光电企业做大做强，主动承接南方光电产业转移，发展印刷电路板、节能发光材料、太阳能、电子电器和电池等产业集群，把鞍山打造成东北光电产业基地。

（三）加快转变发展方式，增强可持续发展能力。切实转变经济增长方式，努力实现经济社会又好又快发展。

优化结构转变发展方式。在做大做强工业的同时，大力发展一、三产业，实现由主要依靠二产拉动经济增长向三次产业共同拉动转变。加快发展第一产业。以设施农业为重点发展现代农业，提高设施农业规模化、标准化、品牌化、产业化水平，打造农产品品牌，培育农产品深加工产业集群。加快发展第三产业。突出发展现代服务业，建设英国乐购广场、大商尚城国际和新玛特购物广场、红星美凯龙家居城、辽宁兴隆大家庭、宁波时代商厦、温州大厦等大型精品商业和法国宜必思、皇冠假日等高档酒店项目；全面推进东北商贸城、北方钢铁大市场、瑞盛汽配城、汽车贸易产业带、北方工程机械大市场建设；积极引进香港海洋广场、卡罗卡国际动漫区等文化旅游产业项目。继续鼓励本地商业银行加速发展，支持鞍山商业银行股份制改造，积极引进广发、浦发、华夏等股份制商业银行入驻鞍山。突出发展房地产业，认真落实国家和省支持房地产业发展的相关政策，城区打造百湖城主题，发展滨湖地产；海城打造滨河主题，推进海西新区、腾鳌新区和牛庄文化旅游区建设；岫岩重点打造洋河水湾主题；台安重点推进旧城改造。实现住宅地产、商业地产和工业地产“三箭”齐发，做到城区、郊区、县域三大空间联动，取得拉动经济、建设城市、改善民生三大成果。

内外并举转变发展方式。积极抢占国内市场的同时，充分发挥鞍山原材料、劳动力等优势，扩大外贸出口。扩大机电产品出口，大力发展装备制造和光电产业，提升鞍山机电产品国际市场竞争力。扩大矿产品深加工出口，积极推进矿产品粗加工向精特深加工转变，提升菱镁、滑石等矿产品出口能力。扩大农产品出口，调整农业结构，发展特色农产品和农产品深加工，积极开拓国际市场。

节能减排转变发展方式。全面推行节能新技术、新材料。继续支持100户重点用能企业实施100个节能技术改造项目，大力推进脱硫工程，严格控制和削减燃煤总量。深入开展辽河流域污染专项整治行动，加快污水处理厂建设。大力发展高新技术产业，实现主要依靠物质资源消耗向主要依靠科技进步促进经济发展转变。建立“官产学研”合作模式，积极引进国外研发团队，收购国外科技型企业，加快科研成果转化，高新技术产值增长20%以上。

（四）发展壮大县域经济，全面推进新农村建设。继续加大对新农村建设的投入。市本级投入“三农”资金12亿元。全年县域地区生产总值和财政一般预算收入增长20%以上。

大力发展县域工业。按照园区、基础设施、主导产业、项目“四位一体”，推进县域工业大发展。突出县域工业区建设。完善县域工业区规划，海城重点推进海城经济开发区、腾鳌经济开发区等园区建设，台安重点推进台安工业区等园区建设，岫岩重点推进雅河、偏岭工业区等园区建设。突出基础设施建设。市政府支持三县（市）各2亿元，完善县域工业区道路、供电、供气、供暖、给排水等基础设施。突出主导产业发展。海城大力打造镁耐火材料、镁金属及深加工、滑石方解石添加剂、钢铁深加工、塑料、五金及灯具产业集群，台安大力打造木业及深加工、建筑起重石油机械产业集群，岫岩大力打造菱镁及方解石深加工、岫玉深加工、中药材深加工和艺术瓷包装瓷及陶瓷原材料产业集群，千山大力打造船舶零部件和金属厨具产业集群。突出项目建设。围绕主导产业，加大招商引资力度，全年县域工业新上项目超过500个，新增规模以上工业企业超过300家。

大力发展现代农业。按照设施农业、加工园区、农产品深加工产业和农产品批发市场“四位一体”，推进现代农业大发展。继续支持设施农业发展，全年新发展设施农业23万亩，设施农业总面积力争达到100万亩。大力发展农产品深加工园区，支持三县（市）各建1个农高区。大力发展农产品深加工产业，支持海城重点发展大蒜、姜等菜产品深加工，支持台安重点发展木产品深加工，支持岫岩重点发展毛皮产品和中药材深加工。加快发展农村大市场，支持三县（市）至少各建1个大型农产品批发市场，打造区域性农产品批发零售集散地。

深化农村改革。全面贯彻落实中央关于推进农村改革发展若干重大问题的决定。完善土地流转制度，健全土地流转服务体系，尊重农民意愿，通过“返租倒包”、经营权入股等形式，实现土地集中经营。创新农村金融服务，积极发展村镇银行、小额贷款公司和担保公司，推行农业保险。完善农村公共服务体系。加快培育农民专业合作组织和农村经纪人队伍。深化集体林配套改革。认真贯彻执行《辽宁省实施<中华人民共和国民族区域自治法>办法》。

（五）全面推进城乡建设和管理，打造宜居城市。继续以创建全国文明城市为统领，努力将鞍山打造成宜居、创业和发展的现代化城市。

加快完善城乡基础设施。加强铁路建设。加快哈大快客、京沈快客（鞍山段）和岫庄铁路建设，规划建设沈辽鞍至海城城际轨道交通。加强公路建设。加快推进丹海高速公路建设。在村通油路基础上，加快推进村屯通油路及村内通油路。加强城市道路和桥涵建设。坚持大街小巷一起修，加快建设二环线，改造人民路、中华路和30条街巷路，完成深营路东线隧道建设及站前过街通道、虹桥改造工程。规划建设城南新区。以海西新区和腾鳌镇改造建设为重点，大力推进全市县城和重点城镇改造。加强电力设施建设。新建1座500千伏变电所、3座220千伏变电所、8座66千伏变电所。加强空港建设。完成腾鳌民用新机场建设和航站楼改造工程，开通直飞北京航线，规划开辟新航线。加强气源建设。推进鞍钢100万立方米煤制气项目，积极引进中石油天然气气源项目。建设10个农村生物质燃气站。加强水源建设。加快建设引汤入鞍二期供水工程和大伙房引水工程，大力改善广大居民饮水质量。

加强城乡生态环境建设。全面加强水系建设。推进南沙河、杨柳河综合整治，重点建设胜利湖、杨柳湖，规划建设千山水库，继续改造海城河和大洋河。加速推进公园改造。新建营城子公园，改造烈士山公园，新增城市绿地100万平方米。继续实施千村绿化和矿山植被恢复工程。大力发展城市集中供热，加强施工扬尘等污染整治。全年城区空气环境质量80%以上天数达到国家二级标准。

全面改善城乡面貌。继续开展专项整治行动。重点治理占道经营、露天烧烤、乱停乱放、乱搭乱建等违法违章行为；加大对噪声、油烟和扬尘整治力度；坚决查处各类毁坏城市市政公用设施的违法行为；规范城市机动车和非机动车管理，打击非法营运。粉饰美化临街建筑，实施站前地区及胜利路、千山中路亮化工程，提高商业繁华区和主干道两侧亮化水平。加强农村村容村貌整治。以鞍羊线、鞍海线、大盘线和海岫路为重点，继续实施镇村环境综合整治。

（六）大力发展社会事业，满足人民群众需求。继续加大投入，全面提升社会事业均衡化、公益化、福利化水平。

加强各级各类教育。继续推进农村寄宿制学校建设，确保每个乡镇拥有一所九年一贯制学校，改造一批农村中小学校危房和城区薄弱学校。进一步提升免费高中办学水平。大力发展职业教育，加快推进职教城建设，把鞍山打造成全国重要的蓝领人才培养基地，提高农村职教生补助标准，对职校部分专业实行免费招生试点。加强高校学科建设，提高办学质量和创新能力。大力支持高校发展，支持辽宁科技大学设立光电产业研究院，为辽宁科技大学解决学生宿舍用地，为鞍山师范学院解决征地动迁问题。加强农村教师队伍建设，适当提高农村义务教育和高中的办学经费，免费定向为农村中小学培训教师。

加强医疗卫生服务体系建设。加快推进医疗资源整合，进一步加强和完善公共卫生服务体系、医疗服务体系和医疗保障体系。加强基层卫生服务体系建设，社区卫生服务中心基础设施全部达到国家规定标准，加快推进乡镇卫生院和村级卫生室标准化建设。采取定向补助方式，为乡镇卫生院配备本科毕业医务人员，为村级卫生室招聘专科毕业医务人员。探索市级公立医院实行药品和医疗器械政府统一采购试点。在全市推行免费婚前医学检查。

大力发展文化体育事业。建立全覆盖的公共文化服务体系。为市级文艺表演团体增配演出车辆和流动舞台，为农民工工地配送电视，做好送戏到乡、到街道、到农民工工地工作。规划建设鞍山大剧院和冶铁博物馆。做好工业遗存和文化古迹等历史文化保护工作。加快建设市奥林匹克体育中心。深入开展群众性体育活动。积极备战省十一届运动会，提高竞技体育水平。

全面提高社会管理水平。进一步强化食品药品质量安全检查，保证市场供应，让人民群众吃上放心食品。补充完善社区老年活动站，提高社区“两站四室”服务水平。强化社会治安防控体系建设，依法打击违法犯罪活动。加强“五五普法”，深入开展矛盾纠纷排查调处工作。做好防灾减灾工作，健全应急管理机制。加强安全生产和消防安全，深入排查治理安全隐患，防止重特大事故发生。

进一步做好人口与计划生育、新闻出版、广播电视、妇女儿童、老龄、民族、宗教、外事、侨务、对台、残联、统计、旅游、接待、史志、档案、气象、地震、人防、国防后备力量建设等工作。

（七）继续为城乡群众办实事，全力造福鞍山人民。认真组织实施八大民生工程，让振兴发展成果惠及广大人民群众。

坚持用发展促进就业，继续做好就业工作。大力开发就业源，深度推进就业、失业实名制。积极开展普惠制就业培训。成立市大学毕业生职业技能培训中心、退役士兵职业技术学校和农民工培训基地，促进各类人员就业和稳定就业。全年实名制就业8万人，城镇登记失业率控制在4.2%以内。

完善社会保障制度，增加城乡居民收入。继续扩大社会保险覆盖面。城镇职工基本医疗保险、城镇居民基本医疗保险和农村新型合作医疗保险实现应保尽保。落实义务教育教师及其他事业单位人员津贴补贴政策，提高企业退休人员基本养老金水平。将已参加城镇居民基本医疗保险的困难企业退休职工转为城镇职工基本医疗保险。完善社会救助体系，进一步提高救助水平，保障困难群众基本生活。继续改造农村敬老院。建设市孤残儿童康复中心。

实施保障性安居工程，多渠道解决群众住房问题。完善住房保障体系，分层次、多渠道解决中低收入家庭住房问题。继续加大危旧房改造力度，加快推进城乡结合部危旧房改造。进一步解决农村困难家庭住房问题。积极推进军队后勤保障社会化，支持驻鞍部队解决在职军官住房问题，切实做好军嫂就业工作。争取建成劳模大厦。

加大资金投入，解决好人民群众关心的问题。做好供暖工作，继续推进铁西等地区供暖分户改造，扩大供暖面积，提高供暖质量。大力发展公共交通，继续购买公交车辆，调整优化城市公交线路；开工建设快客铁路鞍山西站和海城西站，规划建设城市南部和北部长途汽车客运站，新建2个乡镇客运站，方便群众出行。畅通群众诉求渠道，做好信访工作，努力化解各类矛盾，促进社会和谐。

（八）加强政府自身建设，全面提高依法行政水平。要以强烈的事业心和责任感做好各项工作，打造服务政府、责任政府、法治政府。

加强民主法制建设，推进依法行政。主动接受人大依法监督和政协民主监督，坚持向人大及其常委会报告工作，主动与人民政协民主协商。围绕涉及民生、公共服务和社会管理等开展行政立法工作。认真办理人大代表建议和政协委员提案。

加强政府制度建设，规范权力运行。坚持制度用权，严格按照法定权限和程序行使权力、履行职责。继续深化行政审批制度改革，完善公共服务体系，进一步强化对行政审批实施环节的监督，全面优化发展环境。继续加强政府采购中心、政府投资项目招投标中心、土地储备交易中心、房地产交易中心和产权交易中心建设，完善政府采购代购制、政府投资项目代建制。依法全面加强国有资产监管。进一步推行政务公开。

加强机关作风建设，提高行政能力。继承和发扬艰苦奋斗的优良传统，牢固树立过紧日子的思想，加强政府机关和领导干部廉政建设，以人民群众满意为标准，全面打造一支廉洁高效、勤政务实的公务员队伍。全神贯注抓工作，一心一意促发展，用心系鞍山的敬业精神，忘我工作的奋斗激情，求真务实的工作作风，廉洁高效的行政行为，成就伟大事业，开创美好未来。

各位代表，鞍山大发展、快发展的时期已经到来，鞍山老工业基地全面振兴的前景无限美好。让我们以科学发展观为指导，在市委的正确领导下，在市人大、市政协的大力支持下，坚定信心，抢抓机遇，攻坚克难，勇往直前，推动鞍山在新的更高起点上大发展、快发展，向新中国成立60周年献礼，为三到五年再造一个鞍山而努力奋斗！
